# Evidence-Based Identification of Key Beliefs Explaining Adult Male Circumcision Motivation in Zimbabwe: Targets for Behavior Change Messaging

**DOI:** 10.1007/s10461-013-0686-7

**Published:** 2014-01-19

**Authors:** Daniel E. Montaño, Danuta Kasprzyk, Deven T. Hamilton, Mufuta Tshimanga, Gerald Gorn

**Affiliations:** 1 Health and Analytics, Battelle, 1100 Dexter Avenue North, Suite 400, Seattle, WA 98109-3598 USA; 2 Department of Global Health, University of Washington, Seattle, WA USA; 3 Center for Studies in Demography and Ecology, University of Washington, Seattle, WA USA; 4 Department of Community Medicine, University of Zimbabwe, Harare, Zimbabwe; 5 Department of Management and Marketing, The Hong Kong Polytechnic University, Hung Hom, Kowloon Hong Kong

**Keywords:** Voluntary medical male circumcision, Integrated behavioral model, Evidence based demand creation, Behavior change communication, Behavioral theory

## Abstract

Male circumcision (MC) reduces HIV acquisition among men, leading WHO/UNAIDS to recommend a goal to circumcise 80 % of men in high HIV prevalence countries. Significant investment to increase MC capacity in priority countries was made, yet only 5 % of the goal has been achieved in Zimbabwe. The integrated behavioral model (IBM) was used as a framework to investigate the factors affecting MC motivation among men in Zimbabwe. A survey instrument was designed based on elicitation study results, and administered to a representative household-based sample of 1,201 men aged 18–30 from two urban and two rural areas in Zimbabwe. Multiple regression analysis found all five IBM constructs significantly explained MC Intention. Nearly all beliefs underlying the IBM constructs were significantly correlated with MC Intention. Stepwise regression analysis of beliefs underlying each construct respectively found that 13 behavioral beliefs, 5 normative beliefs, 4 descriptive norm beliefs, 6 efficacy beliefs, and 10 control beliefs were significant in explaining MC Intention. A final stepwise regression of the five sets of significant IBM construct beliefs identified 14 key beliefs that best explain Intention. Similar analyses were carried out with subgroups of men by urban–rural and age. Different sets of behavioral, normative, efficacy, and control beliefs were significant for each sub-group, suggesting communication messages need to be targeted to be most effective for sub-groups. Implications for the design of effective MC demand creation messages are discussed. This study demonstrates the application of theory-driven research to identify evidence-based targets for intervention messages to increase men’s motivation to get circumcised and thereby improve demand for male circumcision.

## Introduction

Adult male circumcision (MC) has been demonstrated to reduce HIV incidence among men by up to 60 % [1–4]. MC also offers significant protection from other sexually transmitted infections [5–7]. As a result, the World Health Organization (WHO) and the Joint United Nations Program on HIV/AIDS recommended that MC programs be included as part of the overall HIV prevention strategy in countries where HIV is primarily transmitted heterosexually, and MC prevalence is low [8]. The projected impact of MC programs on HIV transmission and prevalence in countries with generalized epidemics is substantial [4, 9–14] as is the potential long term cost savings from averted HIV treatment costs [15, 16].

In 2007, 13 priority countries in sub-Saharan Africa were identified by WHO for development of MC programs, and a great deal of donor funding has since been directed towards program development and implementation. In order to facilitate rapid scale-up of these programs WHO and UNAIDS as well as other stakeholders developed recommendations, guidelines and tool kits for the provision of services with a largely supply-side focus [17–19]. In addition, WHO coordinated the development of models to optimize the volume and efficiency (MOVE) of MC services. Key features of these models included task shifting, expanded use of less specialized clinicians to perform routine tasks, and bundling of commodities for MC procedures [20, 21]. Despite the significant investment in MC capacity improvements, as of the end of 2012 ten priority countries had achieved less than 20 % of the 2015 targets, and five priority countries where MC is stated to be a priority had reached less than 10 % of their targets [22].

The Ministry of Health and Child Care (MOHCC) in Zimbabwe is implementing a National MC Programme with a goal to circumcise 80 % of adult men by 2015. Modeling estimates showed that 80 % MC coverage could avert 42 % of new HIV infections between 2011 and 2025 [12, 23]. Modeling also demonstrated that more modest reductions in transmission and prevalence as well as cost savings can be achieved with coverage rates as low as 50 % [12]. In Zimbabwe, scaling up to circumcise 80 % of 15–49 year old men by 2015 requires nearly 2 million MCs to be conducted. However, MC uptake has been much lower than desired. A total of about 75,000 adult and teen voluntary medical male circumcisions have been conducted in Zimbabwe since the program began in 2008, about 5 % of the targeted number to be conducted by the end of 2015 [24].

In light of these low rates of MC uptake, there is clearly a need to focus more on the demand creation side of male circumcision, and to develop evidence-based MC communication strategies. Messages designed to motivate men, based on evidence, will maximize the likelihood that men will choose to get circumcised when it is offered, as well as actively seek out MC services. We conducted research in Zimbabwe to identify the beliefs that should be targeted by communication strategies in order to have the greatest potential effect in increasing men’s motivation and uptake of voluntary medical male circumcision.

## Methods

### Theoretical Framework

**Fig. 1:**
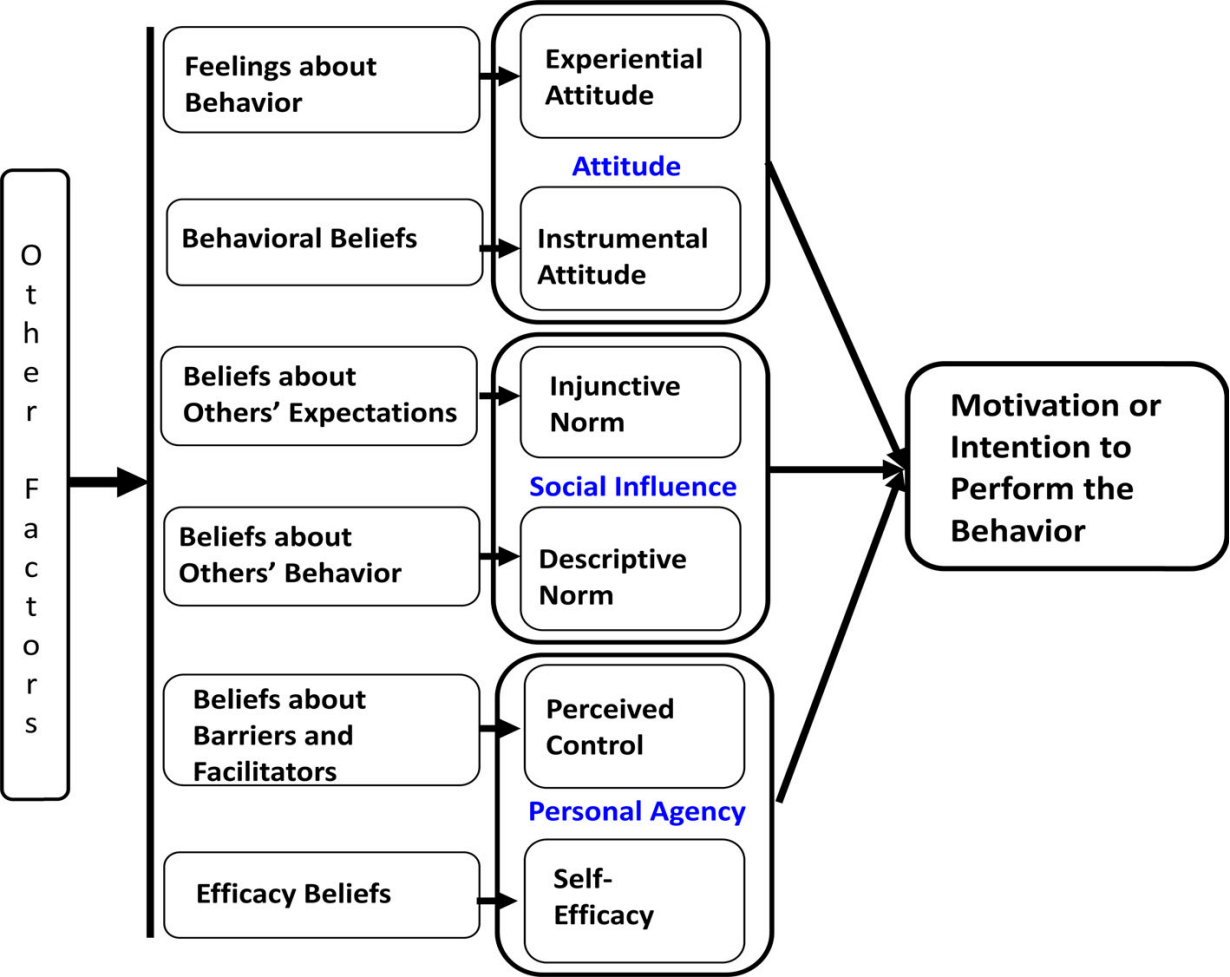
Integrated behavioral model

We applied the integrated behavioral model (IBM) [25–27] to identify the specific key beliefs that best explain men’s level of motivation to uptake MC (see Fig. 1). The IBM includes constructs from several well established theories, including the Theory of Reasoned Action, Theory of Planned Behavior, Health Belief Model, and Social Cognitive Theory [28, 29]. The Integrated Behavioral Model, or Integrative Model [28] is useful not only as a framework to identify issues on which to focus messaging strategies, but also as a strategy to change behavior. Multiple interventions in clinic and community-based settings and meta-analyses have shown the utility of this approach in increasing HIV prevention behavior, including demonstrating effects on both behavioral and biological outcomes [30–37].

Decades of research show that the strongest determinant of behavior is one’s motivation or intention to engage in that behavior [28, 38]. Thus the IBM framework focuses on determinants of intention, consisting of three constructs: attitude, social influence and personal agency [27]. The attitude construct consists of two components. Experiential attitude is one’s emotional or affective response to the idea of performing the behavior. Instrumental attitude is cognitively based, consisting of beliefs about positive or negative consequences or attributes of performing the behavior. Social influence consists of two normative components: (1) beliefs about other’s expectations (injunctive norm) regarding behavioral performance, and (2) beliefs about what others are doing regarding the behavior (descriptive norm). Personal agency consists of two components which impact the ability to engage in the behavior: (1) beliefs about self-efficacy (i.e., perceived ability to overcome obstacles), and (2) perceived control consisting of beliefs about the effect of facilitators and barriers on behavioral performance. Other environmental factors including a person’s characteristics and experiences are considered to impact intention indirectly via these constructs.

This paper and analyses focus on the five belief-based IBM constructs (instrumental attitude, injunctive norm, descriptive norm, self-efficacy, perceived control) because these are most conducive to change. Once the key beliefs (underlying the five constructs) that best explain MC motivation are identified, these key beliefs can be targeted by communications campaigns to change behavior.

### Study Design

The study design consisted of two phases: (1) a qualitative research phase to identify issues with respect to male circumcision among a representative sample of eight key target groups; and (2) a representative sample cross-sectional quantitative survey of members of key target groups. This paper focuses exclusively on the Adult Male target group. All study procedures were reviewed and approved by Battelle’s Institutional Review Board and the Medical Research Council of Zimbabwe.

### Questionnaire Development

A qualitative elicitation study was carried out to design the quantitative survey questionnaire. A sample of 33 men, about equally divided from four urban and rural areas of Zimbabwe, participated in interviews designed to elicit specific issues with respect to each of the IBM constructs. They were asked questions designed to elicit: (1) positive and negative beliefs about getting circumcised, (2) sources of normative influence about getting circumcised, and (3) factors that may make it easier or harder to get circumcised. The interview responses were content analyzed and specific issues were identified with respect to each IBM construct. The content analysis resulted in the identification of 38 positive and negative beliefs about getting circumcised, 21 sources of normative influence, and 14 facilitators and 15 constraints [39].

The quantitative questionnaire was designed based on the qualitative results. The 38 positive and negative behavioral beliefs about getting circumcised were measured on five-point bipolar agree–disagree scales. Injunctive normative beliefs were measured by asking respondents to rate how strongly they agree or disagree that each of the 21 sources of influence would encourage them to get circumcised. Descriptive norm beliefs were measured by asking respondents to rate how strongly they agree versus disagree that each of four sources of influence would get circumcised. Injunctive and descriptive norm beliefs were each measured on five-point bipolar agree–disagree scales. Control beliefs were assessed by asking respondents to rate how difficult versus easy each of the 29 facilitators or constraints make it to get circumcised. These ratings were made on five-point scales ranging from ‘extremely difficult’ to ‘extremely easy’. The 15 efficacy beliefs were assessed by asking respondents to rate how certain they are that they could get circumcised under various circumstances, if they wanted to. Ratings were made on five-point scales ranging from ‘extremely certain I could not’ to ‘extremely certain I could’. Examples of the construct measures are shown in Table 1. Finally, Intention to get circumcised was measured by asking men to rate how strongly they agree versus disagree that they will get circumcised if the MOHCC began a national MC program with MC offered to adult men at no or low cost. This rating was made on a five-point scale ranging from ‘strongly disagree’ to ‘strongly agree’.

**Table 1:** IBM construct belief measures

IBM construct	Measure	Example of question	Response scale				
			−2	−1	0	+1	+2
Instrumental attitude	Behavioral belief	If you were to get circumcised, *it would give you peace of mind*	Strongly disagree	Somewhat disagree	Neither not sure	Somewhat agree	Strongly agree
Injunctive norm	Normative belief	How strongly do you agree or disagree that *your mother* would encourage you to get circumcised?	Strongly disagree	Somewhat disagree	Neither not sure	Somewhat agree	Strongly agree
Descriptive norm	Descriptive belief	How strongly do you agree or disagree that *your closest friends* would get circumcised?	Strongly disagree	Somewhat disagree	Neither not sure	Somewhat agree	Strongly agree
Perceived control	Control belief	*If people describe circumcision as painful*, how much would this make it easy or difficult for you to get circumcised?	Extremely difficult	Quite difficult	Neither/not sure	Quite easy	Extremely easy
Self-efficacy	Efficacy belief	If you wanted to get circumcised, how certain are you that you could get circumcised *if you would be* *attended to by female nurses*?	Extremely certain I could not	Quite certain I could not	Neither/not sure	Quite certain I could	Extremely certain I could

Italicized phrases are examples, and they change for different beliefs that were measured

### Sampling Procedures

The sample of adult males for the quantitative survey was enrolled as part of a community-based recruitment that also included samples of women and parents of adolescent boys for similar surveys about male circumcision. These respondents were recruited using a four-stage probability sampling strategy. At the first stage, we selected four geographic areas in Zimbabwe: Harare, Bulawayo, Mutoko District, and Matobo District. Harare and Bulawayo are the two largest cities in Zimbabwe, with Harare in the Shona ethnic area and Bulawayo in the Ndebele ethnic area. Mutoko and Matobo Districts are rural areas, with Mutoko being primarily Shona and Matobo being Ndebele. Thus, the first stage included urban and rural areas from both main ethnic groups in Zimbabwe. Approximately equal numbers of men were recruited from the four geographic areas.

The second and third stages of sampling were: (1) selection of wards within each of the four geographic sites, and (2) selection of housing units within the wards. Ten wards were randomly selected within each study site. Next, household-based selection and recruitment of men aged 18–30 was carried out within wards until 30 men were successfully interviewed in each ward. Thus, a total of 300 men were to be interviewed in each study site, for a total of 1,200 men overall. Slightly different sampling procedures were applied to housing unit selection in the urban and rural locations due to differences in density and homogeneity of housing units. These different procedures were applied in order to minimize clustering via the possibility of sampling multiple family members. This is particularly important in the rural areas where there is greater family clustering within villages.

#### Urban Sampling

In each of the two urban areas we obtained a listing of all wards, stratified by high and low density. Wards were selected based on density at a rate proportional to the relative density of the population within each ward. In Harare 29 % of wards are low density so we randomly selected 3 (30 %) of the wards from the population of all low density wards. The remaining 7 (70 %) were selected from the high density wards. Similarly, in Bulawayo 18 % of wards are low density so 2 (20 %) of the 10 wards were selected from low density wards while the remaining 8 (80 %) were selected from among the high density wards. Within each of the 10 selected wards in each urban study site, we selected two start points for sampling housing units. Wards were segmented into grid squares, two grid squares were randomly selected, a street was randomly selected within each selected grid, and a house was randomly selected on the street as a start point. After selecting the first house, every sixth house along the same side of the street was selected. If the end of the street or border of the ward was reached, interviewers went a block over and continued the selection of houses along another street, until the target numbers were achieved. The sampling was coordinated in order to select and interview approximately equal numbers of participants across the selected wards, with equal numbers originating from each start point.

#### Rural Sampling

In the rural Mutoko District about 17 % of the population lives in the Mutoko Centre ward. Thus, the sample population drawn from Mutoko Centre was doubled to 60 participants to represent 2 wards, and 8 additional wards were randomly selected from the remaining 28 rural Mutoko wards. In Matobo District there are two semi-urban wards, both of which were selected, while the additional 8 wards were randomly selected from among the remaining 17 rural wards. As in the urban sites, sampling was coordinated to recruit and interview approximately equal numbers of participants across the selected wards. In each of the semi-urban wards the urban sampling procedures were used. In each of the selected rural wards, four villages or settlement areas were randomly selected as start points. Within each selected village or settlement area, the village head or traditional leader was first contacted to obtain permission to conduct interviews. Interviewers next went to the nearest homestead, which may consist of multiple housing units, to select and recruit the first study participant. We then selected the next nearest homestead to recruit another participant, and this continued until the target numbers were reached.

#### Participant Selection within Households

Once a housing unit was selected in either the urban or rural setting the fourth stage sampling procedure was used to select the participant. At each selected household we carried out an enumeration to identify all household members who were male aged 18–30, female aged 18–30, and fathers and mothers of an adolescent boy age 13–17. Household enumeration refusal was less than half of one percent. After completing the household enumeration, we randomly selected one person from the list of eligible individuals. If the selected person was present, recruitment and interviewing procedures were carried out at that time. Otherwise the interviewer arranged to come back, with up to five attempts made to contact and interview each selected person. Only one person was interviewed from each household/homestead.

### Analytic Procedures

Descriptive analyses were carried out on demographic and MC knowledge measures. Mean and standard deviation as well as median and inter-quartile range (IQR) are reported for continuous variables. Percentages are reported for categorical variables (Table 2).

**Table 2:** Respondent characteristics

Characteristic	Harare (*N* = 261)	Mutoko (*N* = 290)	Bulawayo (*N* = 251)	Matobo (*N* = 280)	Total (*N* = 1,082)
Age					
Mean (sd)	22.9 (3.6)	24.1 (3.7)	22.5 (3.5)	22.4 (3.6)	23.0 (3.7)
Median	22	25	22	21	23
IQR	20–26	21–27	19–25	19–25	20–26
Years of school					
Mean (sd)	11.9 (2.2)	10.1 (2.4)	11.3 (2.1)	9.7 (2.2)	10.7 (2.4)
Median	11	11	11	10	11
IQR	11–13	9–11	11–12	8–11	10–11
Monthly family income ($)					
Mean (sd)	642.3 (1284.4)	149.0 (168.2)	439.1 (652.6)	188.6 (266.7)	345 (752.6)
Median	300	90	250	100	170
IQR	150–600	40–200	146.5–490	50–250	65–350
Regularly earn money (%)	54.0	87.9	51.4	65.4	65.4
Marital status (%)					
Married or living as	16.9	41.7	14.4	18.6	23.5
Never married	81.2	54.5	83.6	79.2	74.5
Divorced, separated, widowed	1.9	3.7	2.0	2.2	2.6
Number children (%)					
0	79.5	56.2	81.5	69.2	71.1
1	14.9	29.3	14.8	22.8	20.8
2+	5.6	14.4	3.7	7.9	8.2
Ethnicity (%)					
Shona	90.0	99.7	37.5	8.2	59.2
Ndebele	2.7	0.3	51.8	79.6	33.4
Other	7.3		10.8	12.2	7.4
Religion (%)					
Christianity	92.0	77.9	82.1	71.8	80.7
Other	2.4	3.7	1.2	2.9	2.0
None	5.7	18.3	16.7	25.4	17.3
Knowledge of circumcision (%)					
Surgical removal of foreskin	96.1	80.9	95.2	88.2	88.7
Teaching about sex and STIs	75.3	68.3	61.4	63.2	66.2
Rite of passage	57.4	56.8	40.2	47.1	49.8
Seen/heard MC promotional info from any source (%)	90.4	61.0	91.6	60.4	75.0
Billboard advert	34.1	6.2	39.4	6.8	20.8
TV or radio	64.0	39.3	65.3	30.0	48.9
Flyer	12.3	6.9	19.1	9.6	11.7
Health clinic	11.5	9.7	14.3	9.3	11.1

Our primary analytic goal was to identify IBM measures that best explain MC motivation. We applied an analytic strategy we have used previously and that has been described in prior publications [26, 40–42]. We first carried out internal consistency analysis and computation of Cronbach’s alpha on the questionnaire items used to measure each IBM construct. The construct scores were then computed by taking the mean of the items underlying each construct. Before computing attitude, the behavioral beliefs concerning negative outcomes were reflected so that a higher score was associated with greater disagreement that the outcome will occur, implying a more positive attitude. Attitude toward getting circumcised was then computed as the mean of the 38 behavioral beliefs (Cronbach’s α = 0.92). Injunctive norm was computed as the mean of the 21 normative beliefs (Cronbach’s α = 0.96). Where referents were not applicable the normative belief was coded to zero while all other normative beliefs were coded from −2 (strongly disagree) to +2 (strongly agree). Since many men had either a wife or a girlfriend, these ratings were combined into a single measure. For the few men who had both a wife and girlfriend the measure for wife was used. Descriptive norm was computed as the mean of 4 descriptive norm beliefs (Cronbach’s α = 0.94). Perceived control was computed as the mean of the 29 control beliefs (Cronbach’s α = 0.93). Self-efficacy was computed as the mean of the 15 efficacy beliefs that assessed behavioral certainty under various constraints (Cronbach’s α = 0.94). Once each of the constructs was determined to be internally consistent the IBM model was tested by regressing MC intention on the five computed IBM constructs. The potential impact of clustering by ward and ethnicity were assessed using mixed effects models but no significant clustering was found. A forward stepwise regression procedure was used to assess the association between the IBM constructs and intention. The entry criterion for the regression equation was an F-value with *p* < 0.05.

Analyses were next carried out with the goal of identifying specific beliefs underlying the IBM constructs that best explained MC motivation, and that therefore may be the best focus for intervention messages. For each of the IBM constructs significantly associated with intention, we conducted forward stepwise regression using the beliefs making up the construct as the independent variables. This allowed us to identify the beliefs underlying each IBM construct that provide significant independent contribution to explaining MC motivation. The variance inflation factor was used to check for multicollinearity but no items were found to be entirely subsumed by the other beliefs underlying each construct. After the strongest predictors of intention from within each construct were identified they were combined into a new unique compilation of beliefs. These beliefs were then included as independent variables in a final stepwise regression analysis. This resulted in a final model of key beliefs across the IBM constructs that best explain MC motivation. This analytic procedure was carried out for the complete adult male sample, as well as for subsamples defined by age and urbanicity.

## Results

### Survey Sample Participants

A total of 1,306 men were selected from households, with 52 subsequently found to be ineligible (47 out of age range, 5 previously interviewed in another ward), resulting in 1,254 eligible men asked to participate in the survey. Of these, 53 refused or could not be interviewed after multiple attempts, resulting in an overall survey participation rate of 96 %. A total of 1,201 men participated in the survey, with 300 from each site except Harare where 301 participated. Participation by study site varied slightly from a low of 93 % in Harare to a high of 99 % in Matobo.

### Sample Characteristics

After ensuring that men understood what circumcision is, men were asked whether they were circumcised. Ten percent of men reported that they were circumcised, and the interview was terminated for these men. The remaining 1,085 uncircumcised men continued the survey and were asked the IBM questions to assess factors concerning their motivation to get circumcised. Three of these men did not answer the MC intention question and were excluded from the analysis. Thus, the analyses presented here are for the remaining 1,082 uncircumcised men. Table 2 presents demographic and other characteristics of these survey participants by study site and total.

Participants were on average age 23 with 11 years of education. About one quarter was married, and three quarters had never married. Although half were selected from Shona areas and half from Ndebele areas of the country, 59 % of participants were Shona, 33 % Ndebele, and 7 % of other ethnicity. Seventy-one percent had no children, 21 % had one child, and 7 % had two. The average family monthly income was $345, with 65 % of men reporting that they regularly earned money. Nearly 90 % knew that MC involves surgical removal of the foreskin indicating the success of the awareness campaign. Three-quarters (75 %) reported having seen promotional information about MC. Nearly half (49 %) had seen/heard MC information on TV or radio, while very few (11 %) had seen such information at health clinics. Over three quarters of the men surveyed reported they either somewhat agreed or strongly agreed that MC would protect them from HIV indicating high awareness of the HIV protective benefits of MC.

There was variation in characteristics by study site as expected, associated with urban–rural and ethnic region site locations. For example, participants in the two sites located in the Shona areas are nearly all Shona, while those in the other two sites have a much higher percentage of Ndebele. Those in the urban sites, compared to rural, had higher family income and were more likely to be never married, have no children, have correct knowledge of MC, and have seen/heard MC information from various sources. The Mutoko rural site participants were slightly older and more likely to be married and have children than the other three sites.

### Overall Multiple Regression Results

In the first analytic step to explain MC intention, we used the five computed IBM construct scores as independent variables in the forward stepwise regression. Intention to get circumcised was significantly explained (*R* = 0.71, *df* = 1,079, *p* < 0.001) by attitude (*r* = 0.58, *p* < 0.001; β = 0.15, *p* < 0.001), injunctive norm (*r* = 0.60, *p* < 0.001; β = 0.17, *p* < 0.001), descriptive norm (*r* = 0.59, *p* < 0.001; β = 0.20, *p* < 0.001), perceived control (*r* = 0.61, *p* < 0.001; β = 0.15, *p* < 0.001), and self-efficacy (*r* = 0.59, *p* < 0.001; β = 0.18, *p* < 0.001). Since all five model constructs have highly significant regression weights and zero-order correlations, it appears that motivation for uptake of circumcision is complex, and all constructs may be important potential targets for communication interventions.

Next we examined the beliefs underlying each construct to identify those beliefs that best explain MC intention. Five separate stepwise regression analyses were carried out with the beliefs underlying each model construct as the independent variables. Table 3 lists the beliefs entering each of these regressions along with their beta weights and the zero-order correlations with MC intention. Table 3 also lists the remaining beliefs that did not enter each regression model, and their correlations with MC intention. The results were as follows:

**Table 3:** IBM construct beliefs associated with MC intention (*N* = 1,082)

	r*	[*R* = 0.65] β (*p* value)	Percent strongly agree^a^	
			Not intend MC	Strongly intend MC
*Behavioral beliefs about getting circumcised*				
Will help encourage friends to get circumcised	0.46	0.17 (0.000)	26	76
Will give you peace of mind	0.44	0.15 (0.000)	10	56
Is something that you are too old for now	−0.38	−0.17 (0.000)	24^a^	72^a^
Will give you sense of achievement	0.43	0.10 (0.002)	14	58
Might not heal properly, cause disfigurement	−0.35	−0.07 (0.022)	9^a^	45^a^
Will enhance sexual pleasure for you	0.29	0.10 (0.000)	12	48
Would be against your religion	−0.37	−0.09 (0.003)	37^a^	79^a^
Will result in a slowdown of HIV in Zimbabwe	0.40	0.06 (0.044)	20	69
It may get infected and swollen	−0.34	−0.06 (0.045	12^a^	43^a^
Will make it easier to have sons circumcised	0.38	0.07 (0.021)	31	76
Will cause women to shun you	−0.20	0.07 (0.014)	55^a^	79^a^
Wife/girlfriend may think you will seek pleasure elsewhere	−0.29	−0.07 (0.019)	35^a^	65^a^
Will protect you from STIs	0.37	0.06 (0.043)	17	59
*Behavioral beliefs that did not enter stepwise regression model*				
Procedure would be painful	−0.24			
Wound healing would be painful	−0.24			
May take too long to heal	−0.31			
Doctor may make a mistake and cause you to be disfigured	−0.33			
You may bleed to death	−0.26			
Have to wait too long to have sex	N.S.			
Will protect you from HIV	0.32			
Will still have to use condoms all the time	N.S.			
Will not need to use condoms because protected from HIV	N.S.			
Will be protected from HIV even if condom breaks	0.17			
Will be protected from HIV even if have unprotected sex	0.11			
Means you will live a long and healthy life	0.39			
You will protect your family	0.36			
Means you will not spread HIV to others	0.27			
Penis will be clean and protect you from bacterial infections	0.34			
Will enhance sexual pleasure for your partner	0.28			
Friends may laugh at you and you will be embarrassed	−0.27			
Would be against your culture	−0.35			
Unnecessary because God will protect you from diseases	−0.29			
Would lead you to be tempted to have more sex partners	−0.20			
Would cause you to worry about what happens to foreskin	−0.27			
Pain from previous infections could be reignited	−0.24			
Inappropriate to change the way God created you	−0.31			
You may lose potency	−0.26			
May compromise your sexual performance	−0.25			

	r*	[*R* = 0.61] β (*p* value)	% Agree encourages	
			Not intend MC	Strongly intend MC
*Normative beliefs about who would encourage you to get circumcised*				
Your brothers	0.58	0.24 (0.000)	17	72
Your closest friends	0.52	0.16 (0.000)	14	68
Your culture	0.50	0.16 (0.000)	16	70
People in your community	0.47	0.11 (0.001)	7	51
Your wife	0.59	0.07^b^ (0.033)	4	72
Your girlfriend	0.43		12	59
*Normative beliefs that did not enter stepwise regression model*				
Your family	0.52			
Your father	0.41			
Your mother	0.43			
Your sisters	0.52			
Your uncles	0.52			
Your aunts	0.52			
Your nephews	0.50			
Your grandparents	0.40			
Your religion/church	0.47			
Health care workers in your community	0.38			
Traditional leaders	0.43			
Political leaders	0.39			
The media	0.37			
The ministry of health	0.33			

	r*	[*R* = 0.60] β (*p* value)	% Agree would get MC	
			Not intend MC	Strongly intend MC
*Descriptive norm beliefs about who would get circumcised*				
Your closest friends	0.57	0.23 (0.000)	14	68
Your brothers	0.56	0.17 (0.002)	14	68
Most people like you	0.52	0.11 (0.011)	17	67
Your other male relatives	0.55	0.12 (0.023)	11	61

	r*	[*R* = 0.66] β (*p* value)	% Easy to get MC	
			Not intend MC	Strongly intend MC
*Control beliefs—facilitators/barriers to getting circumcised*				
Availability of equipment and materials	0.55	0.23 (0.000)	30	82
People describe circumcision as painful	0.42	0.09 (0.004)	5	36
If you don’t know how circumcision prevents HIV	0.33	0.10 (0.000)	0	24
If local chiefs/village heads support circumcision	0.52	0.13 (0.001)	23	73
Circumcision is new, not offered before in community	0.41	0.10 (0.003)	2	32
If circumcision is not free to you	0.31	0.09 (0.002)	3	21
If circumcision available in local (including rural) clinics	0.44	0.07 (0.020)	22	70
If circumcision promoted on TV and radio	0.51	0.09 (0.032)	26	74
If you cannot do it privately, so others know	0.36	0.06 (0.021)	9	38
If you did not know where to go for circumcision	0.17	−0.06 (0.033)	3	13
*Control beliefs that did not enter stepwise regression model*				
If your culture was against circumcision	0.33			
If your religion does not accept circumcision	0.32			
If your wife/girlfriend is against circumcision	0.33			
If there were reported cases of complications	0.30			
If circumcision only available at new clinics that only provide circumcision	0.33			
If circumcision only available at city clinics	0.26			
If there was shortage of staff trained in circumcision	0.20			
If you would be attended to by female nurses	0.35			
HIV being in your community	0.30			
If clinic staff explain how circumcision helps prevent HIV	0.48			
If clinic staff explain how circumcision is done and how pain is prevented/reduced	0.44			
If circumcision is promoted in clinics and hospitals	0.49			
If people are assured practitioners are accurate and do not make mistakes	0.47			
If people are assured risk of side effects is very low	0.43			
If you know people who are circumcised	0.45			
Having a clinician in your local clinic do the circumcision	0.41			
Having a specialist do the circumcision	0.45			
If transportation is provided	0.52			

	r*	[*R* = 0.63] β (*p* value)	% Certain could get MC	
			Not intend MC	Strongly intend MC
*Efficacy beliefs—if you wanted to get circumcised, how certain are you that you could if*				
MC is new and has not been offered before in community	0.51	0.17 (0.000)	4	46
MC is available in local – including rural - clinics	0.49	0.24 (0.000)	22	74
Your culture is against it	0.50	0.17 (0.000)	7	51
Your wife/girlfriend is against it	0.49	0.11 (0.001)	10	52
You cannot have it done privately, so others might know	0.43	0.08 (0.008)	7	46
Worried about whether there are adequate supplies in clinics	0.34	0.06 (0.037)	2	18
*Efficacy beliefs that did not enter stepwise regression model*				
If your religion does not accept circumcision	0.49			
If people describe the process as painful	0.48			
If there are reported cases of complications	0.41			
If you do not know exactly how circumcision prevents HIV	0.38			
If it is not free to you	0.40			
If circumcision is only available at new clinics that only provide circumcision	0.42			
If circumcision is only available at city clinics	0.32			
If there was a shortage of staff trained in circumcision	0.30			
If you would be attended to by female nurses	0.44			
If you did now know where to go for circumcision	0.32			

* *p* < 0.001 for all correlations, except *N.S.* not significant

^a^Percent ‘strongly disagree’ is listed for negative behavioral beliefs, in order to consistently list percent with a positive opinion

^b^In order to minimize loss of cases due to missing data, responses for wife and girlfriend were combined into a single variable for the regression analysis, as few respondents had both a wife and a girlfriend

#### Behavioral Beliefs

All but three of the 38 behavioral beliefs were significantly correlated with MC intention. The stepwise regression resulted in 13 behavioral beliefs entering the equation with each providing significant independent contribution toward explaining MC intention (*R* = 0.65). In addition, it is important to ascertain the prevalence of the beliefs held by MC nonintenders and intenders to assess whether there are sufficient proportions of people who may be moved from low to high belief strength by communications targeting the beliefs [41]. Thus, Table 3 lists the percent of MC nonintenders and intenders who strongly agreed with each positive behavioral belief, and the percent who strongly disagreed with negative beliefs. Among the positive beliefs, only a minority (less than 1/3) of nonintenders strongly agreed the outcome would occur, while a majority of strong intenders strongly agreed. Similarly, among the negative outcomes, much higher percentages of MC intenders than nonintenders strongly disagreed with the beliefs.

#### Normative Beliefs

All 21 normative beliefs were significantly correlated with MC intention. MC intention was significantly explained (*R* = 0.61) by five normative beliefs that entered the stepwise regression. Beliefs that MC is strongly encouraged by each of these five normative referents were held by a majority of strong MC intenders, but by fewer than one-sixth of nonintenders.

#### Descriptive Norm Beliefs

All four descriptive norm beliefs significantly explained (*R* = 0.60) MC intention in the stepwise regression. Beliefs that each of the four referents would get circumcised were strongly held by two-thirds of strong MC intenders, but by fewer than one-sixth of MC nonintenders.

#### Control Beliefs

All 29 beliefs underlying perceived control were significantly correlated with MC intention. Ten of these control beliefs entered the stepwise regression, significantly explaining (*R* = 0.66) MC intention. Beliefs that MC would be extremely easy under each of the ten conditions were held by substantially higher percentages of strong MC intenders than nonintenders.

#### Efficacy Beliefs

All 16 efficacy beliefs were significantly correlated with MC intention. Six efficacy beliefs entered the stepwise regression, significantly explaining (*R* = 0.63) MC intention. MC strong intenders were substantially more likely than nonintenders to rate that, if they wanted to get circumcised, they were extremely certain they could get circumcised under each of these six barriers.

#### Final Regression Model

In the third analytic step, we carried out a final stepwise regression analysis to identify the beliefs across the five model constructs that are the strongest in explaining MC intention. We ran stepwise regression, and included all beliefs underlying each model construct found to be significant in the five previous regression analyses. Table 4 lists the beliefs that entered the regression equation. Five behavioral beliefs, two normative beliefs, one descriptive norm belief, three efficacy beliefs, and three control beliefs independently and significantly explain MC intention. Of those items that entered the final model, two behavioral beliefs were positive expectations (‘Will give you peace of mind’; ‘Will enhance sexual pleasure/enjoyment for you’), while three were concerns about negative consequences. Of particular note, no belief about health benefits of MC including prevention of HIV was significant in the final model. Three of the 14 items in the final model specifically related to women. One is the behavioral belief concerned with being shunned by women. It is interesting to note that both the support of a wife/girlfriend and the agency to overcome the objections of a wife/girlfriend were independent significant predictors of intention. Agency to get circumcised despite cultural barriers was also significant. Four of the items in the final model were structural/conditional, with the control belief concerning availability of MC equipment and materials being the strongest predictor of intention among these. Similar to the dual impact of women just noted, perception about MC being new and not previously in the community was independently significant as both a control belief and an efficacy belief. Additionally, “availability in local clinics” was a significant control belief suggesting that the expansion of MC services into local clinics may improve uptake. Finally, the two items with the largest beta weights were the normative beliefs about friends and brothers, suggesting that there is a strong and very personal social acceptance component to MC intention among men’s social networks.

**Table 4:** Final model—overall sample (*N* = 1,082)

IBM construct	Belief	[*R* = 0.74] β (*p* value)
Behavioral beliefs	Will give you peace of mind	0.11 (0.000)
	Something you are too old for now	−0.09 (0.000)
	Will enhance sexual pleasure/enjoyment for you	0.09 (0.000)
	Cause women to shun you and say your penis is different	0.08 (0.001)
	Might not heal properly—cause disfigurement	−0.06 (0.012)
Normative beliefs	Brothers encourage	0.14 (0.000)
	Wife/girlfriend encourage	0.07 (0.018)
Descriptive norm	Closest friends	0.14 (0.000)
Efficacy beliefs	If culture is against MC	0.10 (0.001)
	If MC is new—not offered before in community	0.07 (0.036)
	If wife/girlfriend is against MC	0.09 (0.004)
Control beliefs	Availability of equipment and materials	0.13 (0.000)
	The fact that MC is new, not offered before in community	0.06 (0.028)
	If MC available in local (including rural) clinics	0.06 (0.033)

### Sub-group Regression Results

It is essential to consider whether the beliefs that best explain MC motivation may differ for certain sub-groups of men. This is particularly important in terms of audience segmentation in the design of health messages [43, 44]. We considered sub-groups that could be expected to differ for demographic reasons or that could be targeted in communications campaigns by location. Thus, we decided to compare urban and rural residents since communication campaigns could be different in these settings. We also compared the sub-groups of men aged 18–22 and men aged 23–30 since men in Zimbabwe typically transition from school to adulthood (e.g., getting jobs) between age 22 and 23 and tend to have a main partner or are married by age 24. Thus, it was expected that the MC motivation drivers may be different for men in these different phases of their lives.

#### Urban Men

Intention to get circumcised was significantly explained (*R* = 0.70, *df* = 510, *p* < 0.001) by attitude (*r* = 0.54, *p* < 0.001; β = 0.11, *p* < 0.001), injunctive norm (*r* = 0.58, *p* < 0.001; β = 0.16, *p* < 0.001), descriptive norm (*r* = 0.59, *p* < 0.001; β = 0.27, *p* < 0.001), perceived control (*r* = 0.57, *p* < 0.001; β = 0.12, *p* < 0.001), and self-efficacy (*r* = 0.57, *p* < 0.001; β = 0.19, *p* < 0.001). When five stepwise regression analyses were conducted on the beliefs underlying each model component, a total of 28 IBM construct beliefs were significant predictors of intention (Table 5). Of these, eight entered the final regression model: 3 behavioral beliefs, 1 normative belief, 1 descriptive belief, 2 efficacy beliefs and 1 control belief (Table 6). As in the model for the entire sample population, items specifically addressing the health benefits of MC were not significant independent predictors of MC intention. Two behavioral beliefs were positive expectations (‘Means you will live a long and healthy life’; ‘Will encourage friends to get circumcised’), while one was concern about being too old for MC. The strongest associations were again found to be with expectations related to brothers, close friends, and wives/girlfriends.

**Table 5:** IBM construct beliefs associated with MC intention—urban and rural men

	Urban men (*N* = 512)		Rural men (*N* = 570)	
	r*	[*R* = 0.61] β (*p* value)	r*	[*R* = 0.69] β (*p* value)
*Behavioral beliefs about getting circumcised*				
Will help encourage friends to get circumcised	0.45	0.27 (0.000)	0.46	0.13 (0.001)
Is something that you are too old for now	−0.39	−0.18 (0.000)	−0.39	−0.18 (0.000)
Will give you peace of mind	0.38	0.13 (0.005)	0.47	0.17 (0.000)
Might not heal properly, cause disfigurement	−0.35	−0.14 (0.001)		
Will enhance sexual pleasure for you	0.24	0.10 (0.008)	0.33	0.09 (0.009)
You would worry about what happens to removed foreskin	−0.24	−0.10 (0.017)		
Means you will live long and healthy life	0.33	0.10 (0.024)		
Will give you a sense of achievement			0.48	0.17 (0.000)
May take too long to heal			−0.38	−0.13 (0.000)
Will protect you from STIs			0.45	0.08 (0.031)
Will make it easier to have sons circumcised			0.43	0.11 (0.005)
Would be against your religion			−0.39	−0.13 (0.001)
Is inappropriate because it changes way God created you			−0.32	0.09 (0.022)

	r*	[*R* = 0.60] β (*p* value)	r*	[*R* = 0.63] β (*p* value)
*Normative beliefs about who would encourage you to get circumcised*				
Your brothers	0.52	0.18 (0.001)	0.58	0.32 (0.000)
Your culture	0.48	0.18 (0.000)		
Your closest friends	0.49	0.15 (0.003)	0.55	0.20 (0.000)
Your mother	0.42	0.12 (0.008)		
People in your community	0.44	0.11 (0.017)		
Your wife/girlfriend			0.48	0.13^a^ (0.003)
The media (TV, radio)			0.41	0.11 (0.007)

	r*	[*R* = 0.60] β (*p* value)	r*	[*R* = 0.59] β (*p* value)
*Descriptive norm beliefs about who would get circumcised*				
Your closest friends	0.56	0.23 (0.001)	0.58	0.35 (0.000)
Your brothers	0.54	0.17 (0.011)	0.58	0.26 (0.001)
Most people like you	0.48	0.13 (0.020)		
Your other male relatives	0.54	0.13 (0.048)		

	r*	[*R* = 0.62] β (*p* value)	r*	[*R* = 0.70] β (*p* value)
*Control beliefs—facilitators/barriers to getting circumcised*				
Availability of equipment and materials	0.48	0.22 (0.000)	0.62	0.32 (0.000)
MC is new, not offered before in community	0.41	0.13 (0.006)		
People describe MC as painful	0.41	0.14 (0.003)	0.41	0.10 (0.008)
If circumcision is not free to you	0.29	0.09 (0.019)		
If assured providers accurate/don’t make mistakes	0.45	0.15 (0.010)		
If MC available in local (including rural) clinics	0.37	0.10 (0.017)		
If you don’t know how MC prevents HIV	0.30	0.09 (0.036)	0.34	0.15 (0.000)
If local chiefs/village heads support circumcision			0.60	0.24 (0.000)
If circumcision is only available at city clinics			0.30	0.12 (0.000)
If you did not know where to go for circumcision			0.16	−0.10 (0.004)
If you would be attended to by female nurses			0.38	0.09 (0.018)

	r*	[*R* = 0.62] β (*p* value)	r*	[*R* = 0.65] β (*p* value)
*Efficacy beliefs—if you wanted to get circumcised, how certain are you that you could if*				
Your wife/girlfriend is against it	0.50	0.20 (0.000)		
MC is new and has not been offered before in community	0.48	0.18 (0.000)	0.53	0.17 (0.000)
MC is available in local—including rural—clinics	0.45	0.17 (0.000)	0.53	0.28 (0.000)
Your culture is against it	0.47	0.17 (0.000)	0.52	0.18 (0.000)
If only available at new clinics providing only MC	0.41	0.10 (0.027)		
Worried about whether there are adequate supplies in clinics			0.37	0.10 (0.011)
You cannot have it done privately, others might know about it			0.47	0.10 (0.014)

* *p* < 0.001 for all correlations

^a^In order to minimize loss of cases due to missing data, responses for wife and girlfriend were combined into a single variable for the regression analysis, as few respondents had both a wife and a girlfriend

**Table 6:** Final model—urban and rural men

IBM construct	Belief	Urban men (*N* = 512) [*R* = 0.71] β (*p* value)	Rural men (*N* = 570) [*R* = 0.78] β (*p* value)
Behavioral beliefs	Means you will live long and healthy life	0.13 (0.000)	
	Will help encourage friends to get circumcised	0.11 (0.003)	
	Something you are too old for now	−0.09 (0.014)	−0.12 (0.000)
	Will give you peace of mind		0.13 (0.000)
	Will give you sense of achievement		0.09 (0.020)
	May take too long to heal		−0.08 (0.010)
	Will enhance sexual pleasure/enjoyment for you		0.09 (0.010)
	Is inappropriate because it changes way God created you (suppressor)		0.09 (0.010)
Normative beliefs	Brothers encourage	0.16 (0.000)	0.14 (0.001)
	Wife/girlfriend encourage		0.12 (0.001)
Descriptive norm	Closest friends	0.23 (0.000)	
Efficacy beliefs	If wife/girlfriend is against MC	0.18 (0.000)	
	If culture is against MC	0.10 (0.017)	0.15 (0.000)
	Worried about whether adequate supplies in clinics		0.09 (0.006)
Control beliefs	The fact that MC is new, not offered before in community	0.11 (0.003)	
	Availability of equipment and materials		0.16 (0.002)
	If MC is only available at city clinics		0.09 (0.004)
	If you did not know where to go for MC		−0.09 (0.003)
	If local chiefs/village heads support MC		0.11 (0.029)

#### Rural Men

Intention to get circumcised was significantly explained (*R* = 0.72, *df* = 568, *p* < 0.001) by attitude (*r* = 0.61, *p* < 0.001; β = 0.19, *p* < 0.001), injunctive norm (*r* = 0.61, *p* < 0.001; β = 0.19, *p* < 0.001), descriptive norm (*r* = 0.59, *p* < 0.001; β = 0.14, *p* < 0.001), perceived control (*r* = 0.64, *p* < 0.001; β = 0.18, *p* < 0.001), and self-efficacy (*r* = 0.60, *p* < 0.001; β = 0.16, *p* < 0.001). For the rural men the five stepwise regression analyses conducted for the model constructs found a total of 28 of the IBM construct beliefs to be significant predictors of intention (Table 5). Of these, 14 beliefs entered the final regression model (Table 6). Of the 6 significant behavioral beliefs, all but one (‘Something you are too old for now’) were different from the results for urban men. Three beliefs were concerned with positive expectations of MC, while again (as above) no beliefs had to do with health benefits of MC. There was an additional emphasis on personal agency factors, particularly structural concerns, among rural men. Efficacy and control beliefs about ‘Adequate supplies in clinics,’ ‘Availability of equipment and materials,’ and ‘MC only available at city clinics’ were all independent significant predictors. Additionally, the control belief concerning support of local chiefs and village heads for MC was significant. The role of God also came up as a significant behavioral belief.

#### Men Aged 18–22

Intention to get circumcised was significantly explained (*R* = 0.74, *df* = 538, *p* < 0.001) by attitude (*r* = 0.64, *p* < 0.001; β = 0.20, *p* < 0.001), injunctive norm (*r* = 0.65, *p* < 0.001; β = 0.23, *p* < 0.001), descriptive norm (*r* = 0.60, *p* < 0.001; β = 0.11, *p* < 0.001), perceived control (*r* = 0.66, *p* < 0.001; β = 0.14, *p* < 0.001), and self-efficacy (*r* = 0.64, *p* < 0.001; β = 0.19, *p* < 0.001). For the younger men, the five regression analyses for each of the model constructs found 31 of the IBM construct beliefs were significant predictors of intention (Table 7). Ten of these beliefs entered the final model (Table 8). Two positive behavioral beliefs were significant, and no negative beliefs or beliefs concerning health benefits were significant among this younger group. These younger men where the only group for whom pain emerged as a significant independent predictor in the final model, but as a control belief (personal agency) rather than a behavioral belief (attitude). The role of wives and girlfriends was also less evident among this group, but the support of aunts emerged as a significant normative belief. This suggests that among those who may not have developed a long term relationship with a partner, the opinions and expectations of aunts remains important. Interestingly, paternal aunties and uncles in Zimbabwe served a traditional role in educating adolescents (girls and boys respectively) about sex. Thus it is interesting that even though uncles were included as a normative belief, they did not enter as significant normative influences among these young men.

**Table 7:** IBM construct beliefs associated with MC intention—by age group

	Men age 18–22 (*N* = 539)		Men age 23–30 (*N* = 543)	
	r*	[*R* = 0.70] β (*p* value)	r*	[*R* = 0.61] β (*p* value)
*Behavioral beliefs about getting circumcised*				
Will help encourage friends to get circumcised	0.53	0.27 (0.000)	0.36	0.14 (0.001)
Will give you peace of mind	0.52	0.21 (0.000)	0.34	0.14 (0.004)
Doctor might make mistake, cause you to be disfigured	−0.39	−0.16 (0.000)		
Will protect you from STIs	0.46	0.13 (0.001)		
Will give you sense of achievement	0.50	0.15 (0.000)	0.32	0.08 (0.048)
Would be against your culture	−0.40	−0.09 (0.012)		
Is something that you are too old for now			−0.45	−0.32 (0.000)
Will enhance sexual pleasure for you			0.25	0.11 (0.004)
May take too long to heal			−0.29	−0.12 (0.003)
Will make it easier to have sons circumcised			0.33	0.12 (0.003)
Will cause women to shun you			−0.15	0.11 (0.005)
Wife/girlfriend may think you will seek pleasure elsewhere			−0.28	−0.11 (0.009)

	r*	[*R* = 0.68] β (*p* value)	r*	[*R* = 0.55] β (*p* value)
*Normative beliefs about who would encourage you to get circumcised*				
Your brothers	0.61	0.27 (0.000)	0.48	0.17 (0.002)
Your closest friends	0.56	0.17 (0.001)	0.47	0.13 (0.020)
Your culture	0.51	0.14 (0.002)	0.48	0.19 (0.000)
Your aunts	0.56	0.15 (0.005)		
The media (TV, radio)	0.37	0.11 (0.004)		
Traditional leaders	0.41	−0.13 (0.008)		
People in your community	0.50	0.11 (0.010)		
Your wife/girlfriend			0.46	0.16^a^ (0.002)

	r*	[*R* = 0.61] β (*p* value)	r*	[*R* = 0.58] β (*p* value)
*Descriptive norm beliefs about who would get circumcised*				
Your closest friends	0.58	0.26 (0.000)	0.55	0.29 (0.000)
Your brothers	0.58	0.25 (0.000)		
Most people like you	0.53	0.14 (0.022)		
Your other male relatives			0.55	0.32 (0.000)

	r*	[*R* = 0.72] β (*p* value)	r*	[*R* = 0.61] β (*p* value)
*Control beliefs—facilitators/barriers to getting circumcised*				
Availability of equipment and materials	0.60	0.28 (0.000)	0.49	0.20 (0.001)
People describe MC as painful	0.46	0.13 (0.003)		
If MC only available at city clinics	0.36	0.11 (0.003)		
If local chiefs/village heads support MC	0.57	0.18 (0.000)		
MC is new, not offered before in community	0.44	0.10 (0.022)	0.37	0.10 (0.023)
If circumcision is not free to you	0.36	0.10 (0.011)		
If you did not know where to go for MC	0.13	−0.08 (0.018)		
If MC available in local (including rural) clinics	0.49	0.08 (0.041)		
If your culture was against it	0.37	0.08 (0.046)		
If you don’t know how MC prevents HIV			0.34	0.16 (0.000)
If MC promoted on TV and radio			0.48	0.20 (0.001)
If you would be attended to by female nurses			0.33	0.10 (0.013)
HIV being in your community			0.22	−0.11 (0.007)
If clinic staff explain how MC helps prevent HIV			0.46	0.20 (0.001)
If your religion does not accept MC			0.30	0.09 (0.035)
If clinic staff explain how MC is done and how pain reduced			0.39	−0.13 (0.043)

	r*	[*R* = 0.68] β (*p* value)	r*	[*R* = 0.58] β (*p* value)
*Efficacy beliefs—if you wanted to get circumcised, how certain are you that you could if*				
Your culture is against it	0.54	0.21 (0.000)		
MC is available in local—including rural—clinics	0.52	0.20 (0.000)	0.47	0.25 (0.000)
MC is new and has not been offered before in community	0.54	0.18 (0.000)	0.48	0.16 (0.002)
If only available at new clinics providing only MC	0.49	0.10 (0.022)		
Your wife/girlfriend is against it	0.54	0.10 (0.038)	0.44	0.15 (0.001)
You cannot have it done privately, so others might know about it	0.46	0.09 (0.040)		
You would be attended to by female nurses			0.42	0.10 (0.034)
It is not free to you			0.37	0.09 (0.040)

* *p* < 0.001 for all correlations

^a^In order to minimize loss of cases due to missing data, responses for wife and girlfriend were combined into a single variable for the regression analysis, as few respondents had both a wife and a girlfriend

**Table 8:** Final model—by age group

IBM construct	Belief	Age 18–22 (*N* = 539) [*R* = 0.78] β (*p* value)	Age 23–30 (*N* = 543) [*R* = 0.74] β (*p* value)
Behavioral beliefs	Will give you peace of mind	0.14 (0.000)	0.11 (0.001)
	Will help encourage friends to get circumcised	0.12 (0.001)	
	Something you are too old for now		−0.23 (0.000)
	Will enhance sexual pleasure/enjoyment for you		0.07 (0.023)
	Cause women to shun you and say your penis is different		0.07 (0.027)
Normative beliefs	Brothers encourage	0.13 (0.013)	
	Your aunts encourage	0.12 (0.010)	
	Wife/girlfriend encourage		0.11 (0.006)
Descriptive norm	Most people like you	0.10 (0.014)	
	Your other male relatives		0.15 (0.010)
	Your closest friends		0.11 (0.039)
Efficacy beliefs	If culture is against MC	0.12 (0.001)	
	If MC is new—not offered before in community	0.12 (0.001)	
	If your wife/girlfriend is against MC		0.12 (0.001)
	If it is not free to you		0.10 (0.005)
Control beliefs	Availability of equipment and materials (B)	0.13 (0.002)	
	People describe MC as painful (B)	0.09 (0.011)	
	If MC only available at city clinics (B)	0.07 (0.024)	
	If clinic staff explain how MC helps prevent HIV		0.10 (0.026)
	HIV being in your community		−0.13 (0.001)
	If you don’t know how MC prevents HIV		0.10 (0.005)
	If MC promoted on TV and radio		0.10 (0.045)

#### Men Aged 23–30

Intention to get circumcised was significantly explained (*R* = 0.67, *df* = 540, *p* < 0.001) by attitude (*r* = 0.49, *p* < 0.001; β = 0.10, *p* < 0.001), injunctive norm (*r* = 0.54, *p* < 0.001; β = 0.13, *p* < 0.001), descriptive norm (*r* = 0.58, *p* < 0.001; β = 0.28, *p* < 0.001), perceived control (*r* = 0.54, *p* < 0.001; β = 0.14, *p* < 0.001), and self-efficacy (*r* = 0.53, *p* < 0.001; β = 0.16, *p* < 0.001). For the older men, the regression analyses for each of the model constructs found 29 of the IBM construct beliefs were significant predictors of intention (Table 7). Of those 29 beliefs, 13 entered the final model (Table 8), with more beliefs concerned with wife/girlfriend and sexual pleasure as compared to the younger men. Two positive behavioral beliefs were ‘enhancement of sexual pleasure’ and ‘peace of mind’. For this older group, both normative beliefs and efficacy beliefs related to wives and girlfriends emerged as independent significant predictors of intention. Further, worry about being shunned by women also emerged as a significant behavioral belief differentiating MC intenders from non-intenders. Unique to this group, three control beliefs concerned with HIV also proved to be significant predictors of intention, with awareness of ‘HIV being in community’ and ‘clinic staff explaining how MC helps prevent HIV’ to those who don’t know, being facilitators of MC intention. Not surprisingly, the belief about being ‘too old’ for MC was significant for this older group but not for the younger men.

## Discussion

MC programs in Sub-Saharan Africa have been in the implementation phase since 2007 and much of the focus of these programs has been on supply-side strategies with the expansion of MC capacity. Initial acceptability studies showed possible high acceptance of circumcision to prevent HIV acquisition among men [45]. Quantitative and qualitative studies in Kenya [46, 47], South Africa [48, 49], Zambia [50], Zimbabwe [51, 52] and Botswana [53] suggested MC would be acceptable, provided that the role of MC in HIV prevention was made clear to participants. These studies also found that ostensibly a large proportion of African men (ranging from 45 to 85 %; lowest in Zimbabwe) would choose circumcision if it was safe and low cost or free. It has now become clear that the expansion of capacity has outpaced demand and that initial acceptability did not translate to the adoption of circumcision. Further, building MC capacity alone has clearly proven insufficient to spontaneously engender demand and the ad-hoc efforts to invigorate demand after the fact has met with little success. To date, none of the Sub-Saharan African countries that have implemented a national MC program have been able to reach their target numbers.

Most work to develop communication messages for MC campaigns to motivate men to get circumcised has been based loosely on social marketing principles [54]. Developers have used qualitative focus group discussions or qualitative individual interviews with small numbers of individuals, usually convenience samples. Thus, most communication messages have been designed to target the issues that are most often mentioned by qualitative study participants as potentially affecting motivation. Our research shows that the issues mentioned by most people are unlikely to differentiate those who are motivated from those who are unmotivated. For example, when asked about positive attributes of MC, a high proportion of men mention that circumcision reduces risk of HIV acquisition. Yet, nearly everyone agrees with this belief so it does not explain level of MC motivation nor differentiate men who are motivated to get circumcised from those who are not. Consequently, targeting this belief will likely have little impact in increasing circumcision uptake among men.

Other studies have used quantitative surveys to assess factors preventing non-intenders from getting circumcised. For example, Mavhu et al. [52] carried out a survey of men in Zimbabwe and reported that the main reasons for unwillingness to get circumcised were disbelief that MC protects against HIV, cultural issues, and fear of pain and/or adverse events. However, factors were selected preemptively by researchers and were not assessed based on formative research with men. In addition, respondents only indicated whether the factor affected their motivation, while belief strength was not assessed. More importantly, this was only assessed among MC non-intenders, so it was unclear whether these factors differentiated between MC intenders and non-intenders. Indeed, we found that while men mentioned these behavioral beliefs in our qualitative interviews, protection against HIV, MC being against one’s culture, and pain from MC procedure were non-significant in explaining MC motivation when other beliefs also mentioned were included in the model. However, in contrast, we found that self-efficacy to get circumcised despite cultural barriers, and behavioral beliefs about healing and possible disfigurement, were significant in explaining MC motivation.

Failure of these approaches has led to the need for data-driven evidence-based demand creation through the application of strong behavioral theory to ascertain the appropriate targets to drive the development of communication messages. It should by now be abundantly clear that the content of the message matters, and that if the content is not evidence-based, little behavioral conversion occurs in spite of seemingly high MC acceptability. It is critical to measure beliefs quantitatively in order to determine which beliefs are most strongly linked to motivation, and which ones differentiate men who are motivated from those less motivated to get circumcised. The study presented here clearly demonstrates the application of behavioral theory in quantitative research to identify evidence-based targets for the design of messages to increase MC motivation. This approach may also be applied to other efficacious biomedical interventions.

Fishbein and Cappella [41] described the importance of three criteria for identifying beliefs to target in the development of a behavior change communication program: (a) beliefs should be strongly related to the intention, (b) there should be enough people who do not hold the belief to make the intervention worthwhile in targeting the belief, and (c) it should be possible to change the belief. Our results have clearly identified beliefs underlying each of the IBM constructs that are strongly correlated with MC intention, satisfying the first criterion. With respect to the second criterion, we identified a large number of beliefs that are held by much higher percentages of MC intenders than non-intenders (Table 3). In fact, ten of the 13 behavioral beliefs and all other IBM construct beliefs were strongly held by less than one-third of non-intenders, with most being strongly held by less than 15 %. Conversely, the vast majority of intenders strongly held those beliefs that were most highly correlated with intention to get circumcised. These findings suggest two communication strategies: (1) reinforce issues among men already motivated to get circumcised, to convert them from holding positive intentions to adopting the behavior; and (2) design persuasive messages to change non-intenders’ beliefs to be similar to those of circumcision intenders, thereby leading to increased MC motivation.

Table 3 clearly indicates that the beliefs identified in our analyses meet the first two criteria described by Fishbein and Cappella [41]. The third criterion for identifying beliefs to target is that they must be amenable to change. Not all beliefs are equally susceptible to direct change. Thus, it will be important to select sets of beliefs that will have the greatest impact if they are changed, and that can be targeted in a complementary way. By using stepwise regression analysis we identified beliefs within the IBM constructs that each had significant and independent contributions toward explaining MC intention. It is important to note that these specific beliefs should not necessarily be the only targets for communication messages. Other beliefs that are strongly correlated with MC intention did not enter the stepwise regression due to collinearity with beliefs that had already entered the model. These beliefs should also be considered for intervention, and indeed may be better targets if they are more amenable to change through communication messages. It will be important to target multiple beliefs that are significantly associated with intention as well as beliefs that are highly correlated with a target belief that may not lend itself to direct messaging.

The high correlations between items in the model as well as beliefs that dropped out may be leveraged to generate a broader variety of more effective messaging. For example, one of the strongest predictors of MC intention is the belief that MC will “Give you peace of mind.” This by itself could be an effective target for messaging. But in addition, “peace of mind” is also significantly correlated with both “Prevention of STIs” (r = 0.41) and “Will result in a slowdown of HIV in Zimbabwe” (r = 0.39). Focusing on these incident beliefs may provide additional mechanisms for impacting “Peace of mind” and concordantly, “Peace of mind” may provide a framework for addressing the benefits of circumcision such as prevention of sexually transmitted infections and stemming the epidemic in Zimbabwe in a way that resonates with the target population.

Our analysis of sub-groups by rural–urban and age indicates that there are common significant beliefs across the groups, but there are also important differences. This suggests that mass media campaigns could target the common beliefs, but that messaging must also be group specific in order to have the greatest impact in demand creation. Communication strategy targets may also need to change as uptake and MC demand increases. Thus, monitoring of messaging and the evaluation of their impact will be important to adjust beliefs targeted by communication campaigns.

Design and evaluation of the most effective and complementary sets of messages will require additional research. The next steps in developing an evidence-based MC communication program will involve: (1) designing persuasive messages based on these research findings, (2) integrating those messages into cohesive posters, radio spots, or other small or large media presentation, (3) testing the messages in small groups for appeal, understandability, recall, and persuasiveness, and (4) evaluating the impact of the communication materials on the targeted beliefs and on MC uptake in the community.

Non-surgical MC devices have received substantial attention recently as a means of expanding capacity. At least two such devices have undergone safety and efficacy trials [24, 55–57] and have recently received provisional WHO approval for implementation in Rwanda and Zimbabwe [58]. These devices can be deployed by nurses and thus are expected to have a large impact on capacity for rapid MC scale-up in countries where there are insufficient numbers of physicians to carry out surgical MC for the numbers of men required to reach 80 %. There is a perception that these devices are the solution to achieving scale-up goals. It is important to differentiate logistic feasibility already demonstrated with all the supply-side MOVE model interventions, from uptake motivation. Our research findings suggest that use of the non-surgical devices will have little impact on uptake motivation among men. The devices are designed to avoid surgery and may be perceived to be safer with respect to possible surgical consequences. However, these are not the beliefs that we found to have greatest association with MC motivation. Indeed, it seems unlikely that the use of a non-surgical device will have much if any impact on the beliefs we found to be most strongly associated with MC motivation.

There are at least two important limitations of this study. First, we have assumed that increased MC intention will result in increased probability of MC uptake. Although to our knowledge no studies have been conducted to assess the association between MC intention and action, meta-analyses of diverse behavioral domains have shown a mean correlation of 0.53 between intention and behavior [59]. Additionally, a meta-analysis of effective interventions demonstrated medium to large changes in intention, followed by small to medium effects in changing behavior [60]. There is a clear need to develop strategies to increase this effect on behavior. With respect to MC, it is possible that men with high MC intention may still require additional communication to prompt them to take action. As noted previously, reinforcement of key beliefs to further increase MC intention may be needed to convert men from inaction to seeking MC services. Additionally, a prompt from a key source of influence such as friends or brothers may help convert motivated men to action. These questions concerning how to maximize the effect of increased MC motivation on MC uptake, and whether other prompts may be needed to convert intention to action, need further investigation.

The second limitation of this study is that the results are specific to Zimbabwe. Thus, the beliefs identified as most important to target in communication strategies may not be completely generalizable to other countries. Conversely, the strength of this research is that the study provides a robust framework and scientifically principled methodology to apply in other countries and cultures to identify theory-driven evidence-based beliefs specific to those settings. This may in turn lead to more effective communication campaigns and an increase in circumcision uptake among men who are the targets of MC programs across sub-Saharan Africa.
